# Controlled before-after intervention study of suburb-wide street changes to increase walking and cycling: Te Ara Mua-Future Streets study design

**DOI:** 10.1186/s12889-018-5758-1

**Published:** 2018-07-09

**Authors:** A. K. Macmillan, H. Mackie, J. E. Hosking, K. Witten, M. Smith, A. Field, A. Woodward, R. Hoskins, J. Stewart, B. van der Werf, P. Baas

**Affiliations:** 1 Department of Preventive and Social Medicine, University of Otago, 18 Frederick St, Dunedin, 9054 New Zealand; 2 Mackie Research, Ltd, Auckland, New Zealand; 3 School of Population Health, University of Auckland, Auckland, New Zealand; 4 SHORE Whariki, Massey University, Auckland, New Zealand; 5 School of Nursing, University of Auckland, Auckland, New Zealand; 6 Dovetail Research Ltd, Auckland, New Zealand; 7 DesignTribe Architects, Auckland, New Zealand; 8 TERNZ Transport Research, Auckland, New Zealand

## Abstract

**Background:**

Achieving a shift from car use to walking, cycling and public transport in cities is a crucial part of healthier, more environmentally sustainable human habitats. Creating supportive active travel environments is an important precursor to this shift. The longevity of urban infrastructure necessitates retrofitting existing suburban neighbourhoods. Previous studies of the effects of street changes have generally relied on natural experiments, have included few outcomes, and have seldom attempted to understand the equity impacts of such interventions.

**Methods:**

In this paper we describe the design of Te Ara Mua – Future Streets, a mixed-methods, controlled before-after intervention study to assess the effect of retrofitting street changes at the suburb scale on multiple health, social and environmental outcomes. The study has a particular focus on identifying factors that improve walking and cycling to local destinations in low-income neighbourhoods and on reducing social and health inequities experienced by Māori (Indigenous New Zealanders) and Pacific people. Qualitative system dynamics modelling was used to develop a causal theory for the relationships between active travel, and walking and cycling infrastructure. On this basis we selected outcomes of interest. Together with the transport funder, we triangulated best evidence from the literature, transport policy makers, urban design professionals and community knowledge to develop interventions that were contextually and culturally appropriate. Using a combination of direct observation and random sample face to face surveys, we are measuring outcomes in these domains of wellbeing: road-user behaviour, changes to travel mode for short trips, physical activity, air quality, road traffic injuries, greenhouse gas emissions, and perceptions of neighbourhood social connection, safety, and walking and cycling infrastructure .

**Discussion:**

While building on previous natural experiments, Te Ara Mua - Future Streets is unique in testing an intervention designed by the research team, community and transport investors together; including a wide range of objective outcome measures; and having an equity focus. When undertaking integrated intervention studies of this kind, a careful balance is needed between epidemiological imperatives, the constraints of transport funding and implementation and community priorities, while retaining the ability to contribute new evidence for healthy, equitable transport policy.

The study was retrospectively registered as a clinical trial on 21 June 2018 in the ISCRTN registry: ISRCTN89845334 http://www.isrctn.com/ISRCTN89845334

## Background

Economic development in most cities around the world has been accompanied by a transition from human-powered to fossil fuel-powered transport. The form and function of cities have changed to accommodate motor vehicle dependency, and in many places walking and cycling have been designed out of daily lives. This transformation has had important consequences for human health [1], social and health equity [2] and future urban resilience [3]. New Zealand (NZ) cities follow this pattern: three quarters of urban trips are now undertaken in a private motor vehicle [4].

Road traffic injuries are a major cause of death, disability and health inequity in NZ, with a high rate of road traffic fatalities compared with other Organisation for Economic Co-operation and Development (OECD) countries and a total social cost of over 4 billion NZ dollars in 2017 [5]. Rates are particularly high in children and young people [6]. NZ has recently seen increases in road traffic injury deaths in the past 5 years, even taking into account population growth [7], following a 20-year decline, and despite improvements in regulation, education and vehicle technology. Vehicle-related urban air pollution also has a significant cost in early mortality and morbidity in NZ, including approximately 400 deaths a year [8].

Climate change is widely considered to be this century’s most pressing environmental and public health problem [9]. Transitioning to a low carbon urban future is one of the major challenges facing society. Making a well-planned transition is vital if we are to reap the potential economic, societal and health co-benefits of low carbon cities [10, 11]. Transport is one of the largest and fastest-growing contributors to greenhouse gas emissions. In this sector there are potentially substantial co-benefits for health and health equity associated with carbon-saving interventions [12–14].

Like climate change, obesity is attributable, in part, to the motorization and mechanization of urban lives [15]. NZ has the third highest obesity rates in the world, with 30% of adults classified as obese [16]. Built environments that discourage walking and cycling often associated with physical inactivity, obesity, and related diseases [17].

Car-dependent transport patterns in cities have other undesirable effects on the determinants of health and health equity. These include: socioeconomic, ethnic and gender inequities in access to goods, services, employment and education [2]; threats to social connection and sense of safety from crime [18]; water quality [19]; and household financial vulnerability to expected oil price rises [20]. Very few of these public health outcomes are currently included in transport decision-making.

There are many pathways between urban planning and health and these do not operate independently. Interactions between variables are complex and cyclical [21–23], requiring consideration of the complex causal theory underpinning any intervention study design.

Systematic reviews of interventions to encourage walking and cycling and reduce pedestrian injury have reported that behaviour change programmes alone are largely ineffective [24–27], but infrastructure that improves walkability, traffic calming, and proximate access to destinations including local parks for recreation can all successfully encourage walking and cycling and reduce pedestrian injury [28, 29]. The reviews conclude that there is a dearth of high quality epidemiological research assessing the effectiveness of environmental change, and outline the challenges of such research. Not only are accurate measures of outcomes difficult to achieve, but such studies also require both interdisciplinary research partnerships (including, for instance, epidemiologists, social scientists and built environment researchers), and connections between researchers, urban planning or transport agencies, and communities (*transdisciplinarity*) [21]. Similarly, the growing discipline of EcoHealth suggests that meeting goals to improve human health in the context of social and environmental determinants, environmental sustainability and equity, requires systems thinking; transdisciplinary and mixed methods research; community participation; and approaches that enable rapid translation of knowledge into action [30].

Robust community-level trials of changes to transport are challenging to design and undertake successfully, particularly when the parties involved in the research are not aligned in terms of priorities, funding, timetables and expectations in general. In most instances, natural experiments or quasi-experimental designs have been used. In the United Kingdom [31–33], Australia [34] and NZ [35] this approach has been taken to investigate the impacts on walking and/or cycling of transport agency-led interventions to improve the active transport environment. These studies have found either no, or only small, positive impacts of the interventions on active transport and physical activity in the shorter term, with greater impacts found at longer-term follow-up [32], suggesting that intermediate outcomes as well as physical activity end points need to be considered.

Common limitations of studies of infrastructure for active transport and health include small sample size (and poor generalisability), low response rates, limited follow-up, and lack of control by the researchers over the characteristics, quality or intensity of the intervention. Heterogeneity in the nature of the interventions has limited a clear understanding of optimal infrastructure. Further, there is an absence of integrated outcomes assessment or integrated cost-benefit analysis across broad public health outcomes. There is an absence of evidence about interventions that could reduce social and health inequities mediated by transport system design. Moreover, no studies in this field have objectively measured physical activity [29], despite well-known limitations with using self-reported physical activity. A recent paper on epidemiological bias in natural experiments designed to study the effect of built environment changes on physical activity [32] concluded that even the highest quality studies to date are susceptible to significant systematic error, undermining our ability to draw robust conclusions. The authors proposed study design improvements, including: better matching of control and intervention sites, more stringent adjustment of confounders, use of objective outcome measures, improved reporting of population sample and interventions, and improved measurement of individual exposures to interventions.

Building on the experience of previous natural experiment studies, and attempting to address the challenges identified in the literature, Te Ara Mua-Future Streets (Future Streets) is a *transdisciplinary* project, in other words, it aims to integrate community, policy and research knowledge and assess a range of outcomes [36, 37]. It is an area-level randomised, controlled before-after intervention study, which aims to:

1. develop a best practice walking and cycling infrastructure intervention in a suburb with a high proportion of low income residents (measured by neighbourhood level deprivation) and a high proportion of residents experiencing inequities associated with ethnicity (particularly Māori – NZ’s indigenous peoples, and Pacific peoples);
2. use best practice community co-design for the infrastructure intervention, triangulating community knowledge with high quality evidence;
3. measure behavioural, perceptual and integrated public health outcomes from the intervention; and
4. model the costs and benefits of more widespread intervention implementation.

## Methods

The intervention design was informed by cognitive psychology concepts of affordance, (where the road environment allows for a set of road-user behaviours) [38] and schema and scripts (where people have a mental map of what roads look like and how they work, as well as a set of expected behaviours, reinforced or disrupted by environmental design) [39]. These concepts are incorporated into an approach to road design known as “Self-Explaining Roads” (SER) [40, 41]. The SER approach focuses on three principles: hierarchical road function (for arterial roads, collector roads and local streets); consistency of mass and speed on each road type; and predictable behaviour through consistent design [42]. An earlier SER intervention study demonstrated its effectiveness for reducing motor vehicle speeds and road traffic crashes, as well as changing the way pedestrians used the streets [40, 43].

To achieve our aim of community co-design of an intervention that reduces health inequities, we incorporated a significant community engagement phase and Māori cultural landscape design principles (Te Aranga principles [44, 45]). Māori, the indigenous peoples of NZ, experience significant inequities for many of the health outcomes relating to transport. In addition, Māori have specific rights under their treaty with the Crown (Te Tiriti o Waitangi [46]) for the protection of their wellbeing and equity of outcomes. Low-income and Pacific peoples in NZ also experience significant inequities relating to transport and urban design. We sought to understand whether community co-design with these groups, prioritising Māori, would help to address inequities.

**Fig. 1:**
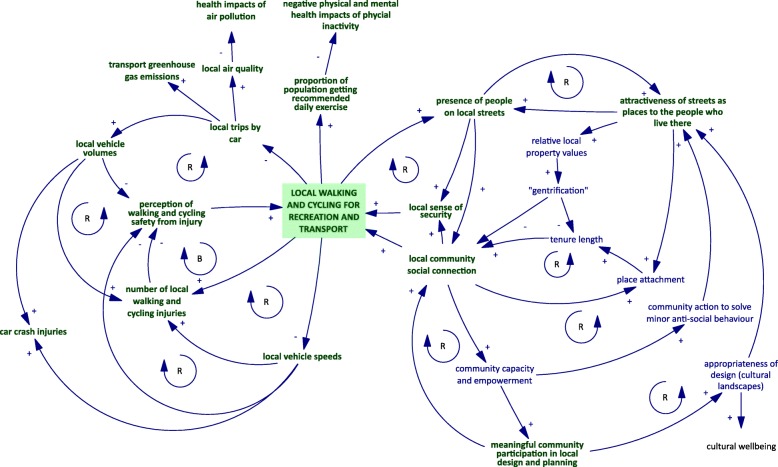
Dynamic causal theory linking built environment, local walking and cycling and outcomes for wellbeing, expressed as a Causal Loop Diagram (CLD). Variables in green are being assessed during the Future Streets study. Arrows with positive signs (+) indicate that a change in the arrow-tail variable leads to a corresponding change in the arrow-head variable. Arrows with a negative sign (−) indicate that a change in the arrow-tail variable leads to an inverse change in the arrow-head variable. R – Reinforcing loop, the result of which is an amplification of the initial pattern of behaviour. – Balancing loop, the result of which is a dampening of the initial pattern of behaviour

We drew on prior ecological causal theories of transport walking and cycling [13, 22, 23, 47–49] to develop a complex, dynamic causal theory, in the form of a Causal Loop Diagram (CLD) [50], of the relationships between infrastructure for walking and cycling and a range of outcomes (Fig. 1). The multiple cyclical feedback mechanisms described in the CLD will vary in strength and relevance by context.

### Research and implementation partnership

The project was developed in partnership with Auckland Transport, the regional transport planning and investments authority, who committed to funding the intervention construction. Project implementation has been guided by 1) a steering group made up of research team and transport agency representatives and 2) a local stakeholder advisory group, led by the local community board, with membership including Mana whenua (Māori tribes with local authority over the Māngere area), community leaders, local primary schools, urban Māori, local Police and health promotion workers. These two sets of partnerships have been active throughout the project. An in-depth discussion of researcher and transport agency relationships is reported elsewhere [51].

### Setting

The project is based in Auckland, the largest and fastest growing city in NZ, with a population of 1.5 million. A program of motorway development and low-density urban growth in Auckland has led to exponential growth in car ownership and use, with a collapse in use of public transport and bicycling as modes of transport [52, 53]. In the most recent national census, private motor vehicles were used for 85% of commutes in Auckland, with public transport, walking and bicycling used less frequently (9, 5, and 1%, respectively) [4].

In 2013, the following criteria were used to choose intervention and control study neighbourhoods, of approximately 1400 households and 6500 people in each neighbourhood:

- **Primary characteristics**

1. Potential for accessibility to local destinations, drawing on existing indices of accessibility and walkability at a census area level [54, 55]
2. High levels of social and economic deprivation as measured by the census-based neighbourhood deprivation scale (NZDep2006 Index of Deprivation [56])
3. A high proportion of Māori and Pacific residents
4. Higher than average rates of road traffic injury

- **Secondary characteristics**

5. Based on natural ‘community’ boundaries, without major dividers such as a motorway
6. Alignment with the region’s cycle network planning, so that the intervention area is likely to eventually connect to a wider network

**Fig. 2:**
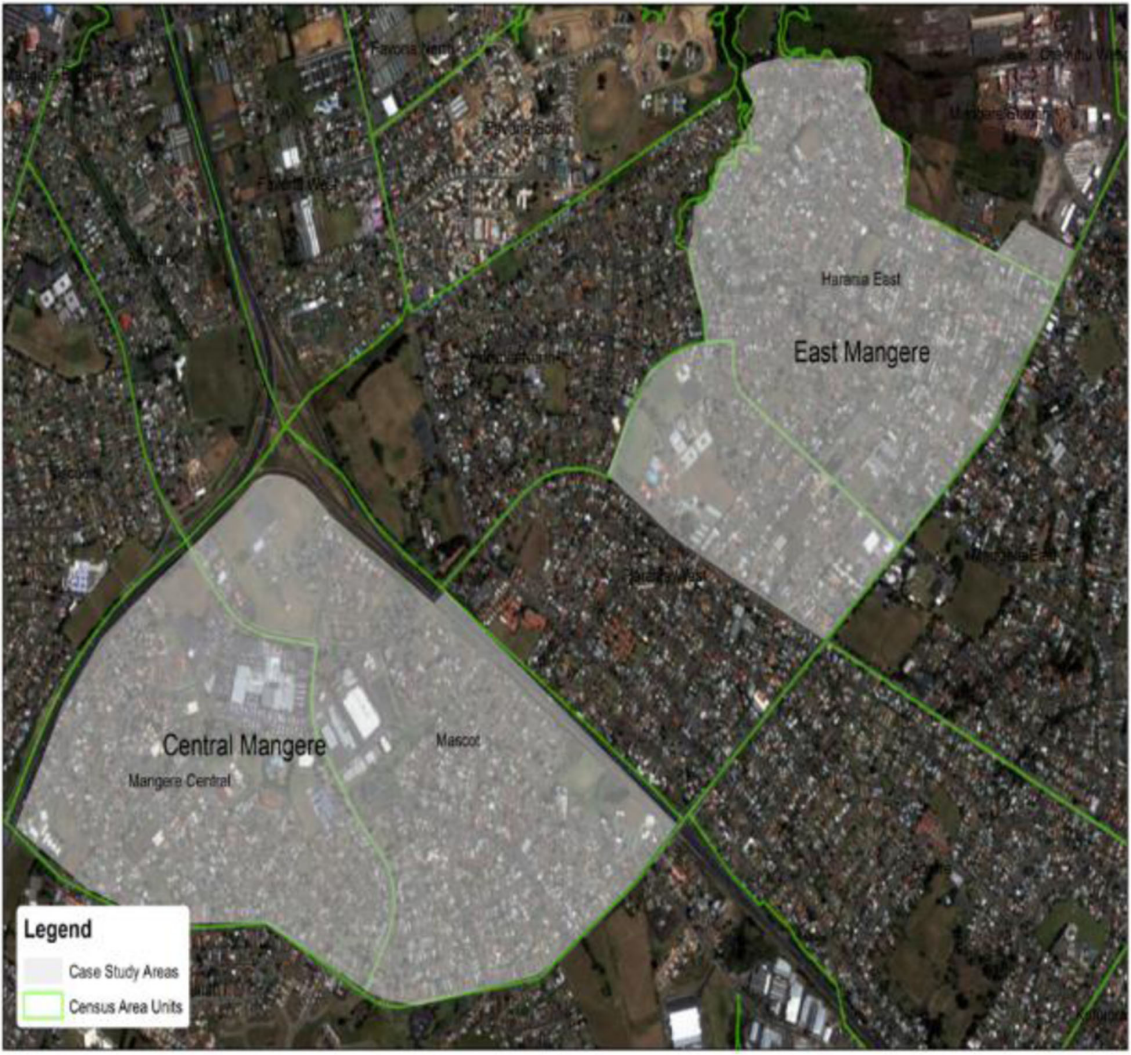
Map of intervention and control areas. Intervention area is labelled “Central Māngere”, control area is labelled “East Māngere”. (Map credit: Philip Donovan, Massey University 2013, with permission)

Two neighbourhoods within the larger suburb of Māngere were selected by the steering committee in 2013. For the purposes of the project, these areas were named ‘Māngere Central’ and ‘Māngere East’. These areas are shown in Fig. 2. At the time of selection, the two areas had comparable demographic profiles and street layouts but are separated by a motorway. Auckland Transport had identified no major infrastructure projects planned in either area during the study period.

A computerised random number generator was used to randomise the two areas to intervention and control. Since there were only two areas, random allocation could not serve its usual purpose (to distribute evenly known and unknown confounding factors), rather it ensured the intervention area was not chosen because it was more in need of improvement or more receptive to change.

Intervention and control areas were both to receive ‘business-as-usual’ transport initiatives (including other minor infrastructure improvements, educational and promotional activities) during the project. These included active transport encouragement or organisational travel planning that might be occurring at a regional level. Any differences in delivery of such initiatives across the study sites are being documented.

### Intervention design

A detailed description of the process and outcomes of the participatory intervention design has been reported elsewhere [57]. Contextual transport and place information from community groups and Māori iwi (extended kinship group) with local area authority was triangulated with baseline vehicle counts and speeds, pedestrian and cyclist counts, and video movements of pedestrians and cyclists across the intervention and control areas, to identify most used routes and crossings, as well as places of high conflict between road users. A clear road hierarchy map was developed from these data, differentiating three types of road: arterial roads, collector roads, and local streets.

These multiple datasets were used to develop a set of guiding design principles that prioritised the issues identified through the participatory process. Initial draft infrastructure intervention designs were then developed by Auckland Transport, including a range of infrastructure changes to reallocate road space from vehicles to pedestrians and cyclists; improve street crossing safety and convenience; improve the safety of routes through parks; and landscaping to reflect indigenous culture and history.

**Fig. 3:**
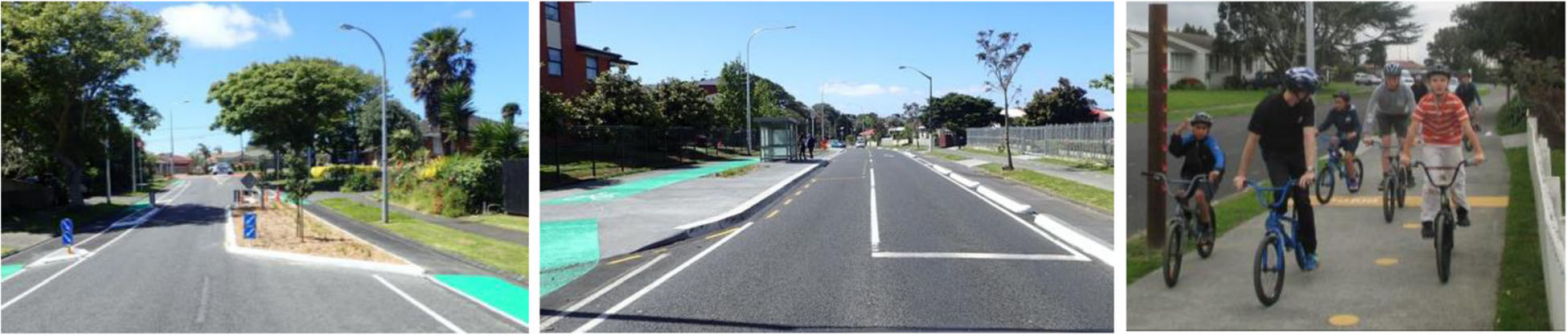
Examples of intervention infrastructure. A demonstrates physically separated cycleways, road narrowing and indigenous planting on a local street previously being used like an arterial road. B demonstrates physically separated cycleways, widened and smoothed footpaths and treatment of cycleways around a bus stop, on an arterial road. C shows a greatly widened and smoothed shared footpath/cycle nursery, along with indigenous markings (marker posts and paintwork) on a local street, as part of a fitness circuit (Photograph credits: Richard Scott, Mackie Research 2017 and Stuart Houghton, Boffa Miskell 2017, with permission)

An iterative process of engagement and revision was used to develop the final designs, examples of which are shown in Fig. 3.

### Outcome measures

Measures of hypothesized influences on, and outcomes of, walking and cycling are described below. The outcome measures are listed in Table 1. Short-term follow-up occurs 6–14 months following intervention completion, and longer-term follow-up will occur 2.5–3 years post intervention. Outcome measures include physical health outcomes (e.g. diabetes, injury), risk factors (e.g. physical inactivity and air pollution), social and environmental health outcomes (e.g. social connection), with a mixture of individual and area-level measures. On the basis of travel behaviour and risk factors we will model longer-term health outcomes that cannot feasibly be measured in a study of this duration. Follow-up measures are matched by month to baseline, to control for seasonal effects. Daily weather data will be included as a control variable in analyses.

**Table 1:** Summary of Future Streets outcome measures and their timing

	2014	2015	2016	2017	2018	2019	2020	2021
*Intervention construction*		*X*	*X*					
Objective physical activity	X						X	
Road traffic injury	X					X		
Air quality measures	X					X		
Diabetes risk	X			X		X		
Vehicle speeds	X			X	X	X		
Road user counts	X			X	X	X		
Video measure of road user behaviour	X			X	X	X		
Face-to-face survey	X			X		X		
Climate pollution modelling	X			X			X	
Qualitative interviews/focus groups	X				X			
Retrospective analysis of safety and security		X						X
Health outcome and cost-benefit modelling					X		X	X

Because of the wide range of study outcomes and the paucity of previous intervention research in this area, calculating a sample size for the outcome measures was not straightforward. We were particularly interested in the objective measurement of physical activity, as a novel addition to the existing literature, and calculated sample size based on changes in steps measured by pedometry. Repeated measures sample size was based on the ability to detect a 1000 steps per day change in physical activity measured by pedometer, supported by previous research [38], with the expectation of 30% loss to follow up for survey participants. Based on these calculations, we aimed to recruit 360 child and 720 adult participants (to allow age group sub-analysis in the adult group) in each area to the survey and pedometer measures. This allowed for 80% power to detect change with a significance level of *p* < 0.05.

### Random sample face-to-face resident survey

A longitudinal random sample survey of children and adults, combined with pedometer measures of physical activity, is one of the core components of the study. We visited all houses in the case and control areas in 2014 to enumerate households so that we could sample participants by person and avoid household cluster effects. Individual adults and children were randomly recruited across the two areas using a pre-determined probability of selection, different for adults and children, based on the latest census population data and our expected response rate. Face to face surveys were conducted with the recruited participants (adults and parent-child pairs), using their language of choice (Samoan, Tongan, English). All households with a participant in 2014 were revisited in 2017 and those who took part in the 2014 survey were invited to repeat the survey. A replenishment sampling strategy was used to replace participants who were lost to follow-up, by recruiting new participants from the original address, using the same overall individual selection probability as in the sample of 2014. This sampling strategy was designed to retain as much of a longitudinal sample as possible, despite high residential mobility in Māngere. The replenishment sample at short-term follow-up will become a longitudinal sample at longer-term follow-up. Replenishment samples are useful [58] as they may increase power in analysis; enable exploration of the randomness of loss to follow-up, assess changes in the population, and will facilitate recruitment of a new group of children to account for aging. In 2019 this strategy will be repeated.

The survey contains the following elements:

- **Demographics**: age groups; ethnicity, sex, duration of residence in Māngere, marital status, highest academic qualification, current employment situation; whether they were forced to buy cheaper food or visit a foodbank in the last 12 months (and if so, how frequently) [59]; the number of driveable motor vehicles and usable bicycles at the dwelling; individuals in the household holding a NZ drivers licence; whether the dwelling is rented/owned; combined household income before tax in last 12 months; and household composition (children, youth, adults). Questions were adapted where possible from the NZ Health Survey [60] and the 2006 Census [61].
- **Active transport**: items from the iConnect survey [62, 63] were used to enumerate trips to and from destinations across seven categories of trip purpose (e.g. work, study, or social activities). For each category, participants were asked to report the total number of journeys in the previous 7 days, average trip time, and main transport mode used.
- **Leisure-time physical activity**: items from the International Physical Activity Questionnaire-Long Form (IPAQ-LF) [64], previously demonstrated to be comprehensive, reliable and valid, were used with the previous 7-days as the reference period. The IPAQ-LF provides separate estimates of physical activity in specific domains.
- **Sitting time:** assessed using the IPAQ-LF. Sitting items ask respondents to report usual duration of sitting while at work, at home, while doing course work and during leisure time in the last 7days.
- **Neighbourhood perceptions and social wellbeing:** individual items on neighbourhood perceptions were drawn from the validated, reliable Neighbourhood Environment Walkability Scale-Abbreviated version (NEWS-A) [65]. Perceived ease of cycling in the neighbourhood was assessed using an item from the Australian Cycling Connecting Communities Study, in which this item was associated with residents wanting to ride more, and was predictive of past cycling in previous studies [66, 67]. Perceived neighbourhood safety, social cohesion, and social connection were assessed using items from the Ranui Action Project Survey, successfully used with adults and children from ethnically diverse low-income neighbourhoods [68], and to understand children’s independent mobility in Auckland [69].
- **Self-reported road traffic injuries (RTIs):** questions from the World Health Organization (WHO) guidelines [70] for conducting community surveys on injuries have been adapted to elicit self-reported RTIs as well as non-injury collisions and crashes for all modes of transport in the past year, counting injuries which prevented carrying out normal daily activities for at least 1 day or for which they had any type of treatment. For each injury, information was collected on health service utilisation, transport mode at the time of injury, other vehicles involved, date, and location.
- **Physical abilities**: questions about hearing, vision, mobility, and wheelchair or mobility scooter use are based on the 2006 NZ Disability Survey [71].

### Objective physical activity measurement

At the time of the baseline survey, all survey participants were invited to take part in pedometer measures of physical activity. Consenting participants wore a sealed Yamax Digiwalker CW300 pedometer (Yamax Corp., Kumamoto, Japan) for the next seven consecutive days. These pedometers are valid and reliable for measuring steps in adults [72, 73] and children [74], are low cost and simple to use [75]. In 2019 we will invite all survey participants to repeat an identical 7-day pedometer protocol.

### Traffic and road user measures

At baseline and short-term follow-up (seasonally matched), traffic volume and speeds have been measured with tube counters over 7-day periods, in 8–10 locations per area. Simultaneously, pedestrian and cycle counts and road user interactions have been measured using 12-h video monitoring over 2 week days and two Saturdays. We will repeat location- and season-matched measures in 2019. A tested coding framework will be used to categorise road user interactions.

### Air quality measures

Between November 2014 and February 2015, avoiding school holidays, we undertook passive Nitrogen Dioxide (NO_2_) sampling at 28 sites in each of the intervention and control areas using Palmes diffusion tubes, a method commonly used for cost-effective air quality monitoring in NZ. NO_2_ was chosen as it is a sensitive and specific indicator of traffic-related air pollution. Sampling was repeated three times to minimise the impact of unusual traffic and weather events, and duplicate sampling was undertaken to minimise lost and outlier data. Sites for monitoring were matched to video and speed monitoring sites, with further sites representing high exposure, and some randomly allocated. Local meteorological conditions were recorded from the Māngere meteorological site. In 2019 the same monitoring protocol will be repeated, matched by date to the baseline monitoring to avoid seasonal bias.

### Population-level glucose regulation and diabetes risk

We will examine whether the intervention is associated with a shift in the population distribution of glycated haemoglobin (HbA1c) compared to the control group by obtaining an anonymised dataset of HbA1c tests taken from residents in the study areas before and after the intervention. HbA1c is considered the optimal epidemiological measure of glucose control at a population level [76], and is strongly associated with cardiovascular risk [77, 78]. In NZ, HbA1c testing is part of cardiovascular risk assessment for all men aged ≥45 years, women aged ≥55 years, and high risk groups including people with a Body Mass Index over 30, Māori, Pacific, Indo-Asian people, those with a family history of diabetes, and women with a history of gestational diabetes [79]. For those with diabetes or prediabetes the recommended frequency of HbA1c testing is at least once every 6–12 months [79]. The prevalence of diagnosed diabetes in Māngere is about 15%, and about 30% of the population meet the criteria for prediabetes [80]. The expected level of adult HbA1c testing for any year is therefore high (we estimate approximately 50%). We will match National Health Index (unique identifier) numbers over time to achieve a longitudinal sample of tests for residents of both areas. This sample of tests will include those with ‘normal’ glucose metabolism, those considered to have prediabetes (HbA1c 41–49 mmol/mol) and those with diabetes (HbA1c ≥50 mmol/mol) [79]. Accounting for increases in HbA1c with age we will chart population distributions of HbA1c, and analyse changes in mean HbA1c between areas.

### Traffic crash analysis

Survey participants have been invited to provide consent for their details to be linked to several routinely-collected data sets: the NZ Transport Agency Crash Analysis System [81] (NZTA CAS), Accident Compensation Corporation data, and the Ministry of Health National Minimum Data Set and Mortality Collection. Collectively, these datasets provide the most comprehensive data on RTIs, and avoid problems with incomplete recall of injuries by participants [82]. About 80% of baseline survey participants consented to data linkage. We will use these data to compare injury rates before and after intervention. We will also undertake analysis of routinely collected crash data at an area level using the NZTA CAS. This area-level analysis will not be restricted to survey participants or residents, as it will record all crashes and injuries occurring in the intervention and control areas. CAS generates data on crash location, severity and demographic variables. As they are rare events, pre-post changes in reported crash and injury numbers will be analysed using 5 years of pre- and post-intervention data.

### Objective safety and security scores

Previous research about neighbourhood safety and aesthetics has found mixed associations between subjective and objective measures, and called for further research measuring both [83, 84]. We undertook fine-grained street segment objective analysis at baseline using the validated NZ Systematic Pedestrian and Cycling Environment Scan audit tool [84] and Google Street View (updated every 1–3 years) in keeping with previous virtual audit research [85]. We will repeat the objective scoring of street segments in 2021, using Street View data closest to the time of the post intervention surveys. Using the same protocol, we will analyse correlation between post-intervention subjective and objective scores, and test whether there has been a significant change in either or both as a result of the intervention.

### Qualitative interviews and focus groups

In the intervention area only, face-to-face qualitative interviews were undertaken pre-intervention and early post-intervention with eight key informants who lived or worked in the neighbourhood. Interviews were conducted seated while viewing an area map, or while walking selected neighbourhood streets (‘Go-Along’ interviews [86]). Walking routes were mapped and, where relevant, photographs taken by interviewees. Semi-structured questions were used to explore perceptions of place, access to local destinations, and enablers and barriers for active transport and use of local public spaces.

In the intervention area focus groups were conducted in two primary (elementary) and one secondary school with children and young people aged 9–10 and 14–15 years, respectively. Children and young people were asked to show on area maps the routes they take to school, local shopping and recreation, places and streets they felt safe and unsafe with reasons, their mode of travel to school, local places they play and any issues locally that prevent or support them in active travel.

Data from these interviews and focus groups contributed to the intervention design. Repeat interviews and focus groups have followed the completion of the intervention, to explore residents’ perceptions of the intervention effects and add meaning to the quantitative effect measures. Thematic analysis will be used to analyse the focus groups and interviews, applying both deductive (using themes based on our causal theory) and inductive approaches.

In both the intervention and control areas, a record of major events and changes relevant to the study outcomes will also be constructed, with the aim of qualitatively assessing their impact on study outcomes.

### Statistical analysis

Data will be analysed according to area, the scale of measurement, distributional assumptions and sampling structure, accounting for the longitudinal sample as well as the cross-sectional sample. We will use separate generalised linear mixed models, adjusting for repeated measurements. If the replenishment sample is comparable to the participants lost to follow-up, we will use both sets of data in combined models, testing the sensitivity of results to our assumptions of similarity. Potential explanatory model terms and covariates in the statistical analysis will be identified a priori from our causal theory and by the use of Directed Acyclic Graphs. Because this is a complex, area-level intervention, with social components, residents living in the intervention area will be considered “exposed”, while residents living in the control area will be considered “unexposed”. In our primary analysis, we will consider exposure to be homogenous across the areas. Secondarily, we will be exploring more nuanced approaches to individual-level intervention exposure, including distance between residential address and the nearest intervention treatment.

Outcome variables to be included in analyses include: mean, median and 85% vehicle speeds, and vehicle counts by road type; changes in the mean numbers of pedestrians and cyclists seen in video data; mean reported minutes walked or cycled; mean trips walked or cycled; median weekly minutes of reported physical activity - total, recreational and transport; mean steps per day as measured by pedometer; mean perceived and objective sense of security and neighbourhood perceptions scores; self-reported and routinely recorded injuries by mode and by injury severity; changes in mean NO_2_ on different route types; and changes in mean HbA1c. Some identical questions between child and adult surveys will be analysed together, though we will also analyse child and adult survey data separately, stratify survey analyses by sex, adult age group, income, and ethnicity (including separate Pacific ethnicities), and explore differences by mobility.

### Integrated health outcomes and economic cost-benefit modelling

Our existing system dynamics modelling [13] focuses on cycling, extrapolating health and economic outcomes from changes in transport mode share and resulting physical activity, air pollution, injury, and greenhouse gas emissions. The findings from longer-term follow-up of Future Streets will enable us to refine the effect size assumptions in the model, include influences on walking, and extend the range of outcomes to include those relating to diabetes, sense of security and social connection.

We will model the theoretical impacts of wider regional or national implementation of Future Streets on population health, including, where possible, on health equity by income and ethnicity. We will use up to date economic values for outcomes to undertake cost-benefit analysis in keeping with methods already used in the transport sector [87]. We will explore the equity impacts of different scenarios for wider implementation of the Future Streets intervention. Scenarios may include: business as usual (based on current implementation patterns, which may include ‘squeaky wheel’ and ‘ad-hoc’ approaches); random (in which all areas have an equal likelihood of receiving the intervention); needs-based implementation (in which populations with the highest ‘need’ for the intervention receive additional priority); and proportionate universalism [88], in which disadvantaged social groups with lower health status receive additional priority.

## Discussion

Future Streets builds upon previous natural experiment studies assessing the impacts of transport physical environments on a range of health, social and environmental outcomes [32, 33, 35]. However, it has a number of unique strengths, including the objective measurement of a range of outcomes including physical activity. The focus on assessing interventions that may work to reduce health and social inequities is a new addition to the existing literature. The study’s controlled before-after intervention design has given the research team significant levels of control over the intervention design, area selection and implementation. The intervention was designed at an area level rather than as isolated pieces of infrastructure spread thinly over larger areas. The partnership between the research team, the community and transport planning and funding authorities meant we have been able to achieve design, funding and implementation of a transport infrastructure intervention within the constraints of a research project. The participatory intervention design brought best evidence from the literature together with crucial, context-specific community knowledge about barriers, desire lines and destinations, and local design knowledge, to develop an intervention design that has the best chance of achieving change. Control over area selection meant we have been able to match intervention and control areas on important potential confounding factors, including urban design and demographic factors.

The cross-disciplinary research team has been critical to the strong relationships with community groups and transport planners and funders, while also enabling us to develop an integrated, mixed methods plan for assessing a broader range of outcomes than has been possible in similar studies to date. In addition to breadth and comprehensiveness of outcomes, the combination of objective and subjective measures is likely to strengthen the existing evidence, and will enable triangulation between subjective and objective measures and improve validity. The broad range of outcomes will enable a comprehensive public health benefit-cost analysis of wider implementation of the intervention, rare for transport interventions.

Despite these strengths, there are significant challenges and limitations to the study. The major risks of tying a large research project to a process of design, funding and implementation of a significant amount of transport infrastructure by a local government agency, along with the challenges of this kind of collaboration, have been described already [51]. Uncertainties about the level of infrastructure funding, ability to innovate and timing of construction made it difficult to implement the study as first planned. Gradual release of funding over the period of construction meant that additional, although modest,changes to the street infrastructure have continued beyond the official date of completion. A 12-month delay in construction meant that the period between intervention completion and the first survey follow-up has been shorter than planned, and the delay is likely to have increased loss-to-follow-up among survey participants. Funding and planning limitations meant we were only able to include two areas in the study, compounding the limitations of a clustered study design. Identifying two areas that were closely matched on confounding variables meant they were close together geographically, increasing the risk of contamination, despite their severance by a motorway. Future Streets is necessarily a “realist” epidemiological study, where a balance is necessary between the recommendations of epidemiological theory, the funding available, the constraints of the transport agency and the priorities of the community.

The complexity and context-specific nature of the intervention itself make it difficult to implement elsewhere in precisely the same form. Both the process of community engagement and the physical infrastructure may play a part in any outcomes realised, while the intervention itself comprises a mixture of physical changes designed specifically for the Māngere community. We have focused on suburb level, which means that any shift from car use to active transport as a result of the intervention may be more likely to be for shorter trips within the suburb (such as trips to primary school, recreation facilities, and local shops), rather than longer habitual trips to work and higher education, although improvements in infrastructure for active transport have also improved public transport accessibility. Currently, the Future Streets cycling infrastructure is not well connected to a wider cycle network that would enable longer trips, although one early outcome of the intervention is the catalysis of discussions about wider linkages. In the meantime, this constraint may limit the size of effects across all the outcomes of interest.

Future Streets has a clustered study design, with the intervention acting at an area level, potentially undermining assumptions about observation independence and causing bias. Potential issues caused by intra-cluster dependencies include: self-selection on variables that affect outcomes, inherent differences between clusters, and external influences on one cluster but not others. In the case of Future Streets, it is unlikely that self-selection related to influences on walking and cycling have occurred before the intervention, and the intervention and control areas are well-matched on demographics, walkability and accessibility. However, with residential mobility it is possible that self-selection will be become an increasing issue as the study progresses. We have worked closely with Auckland Transport to ensure, as far as possible, that other external walking and cycling influences (especially encouragement measures) are implemented evenly across the intervention and control areas. However, it is impossible in a study like this to control all external transport factors. In addition, the intervention itself can be considered to act in two ways – through influences that are strictly individual, and through others that are “infectious” and social, including the way the infrastructure stimulates community-level activities encouraging walking and cycling. This means that controlling for intra-cluster dependencies in the analysis, while potentially reducing error, could under-estimate the true effect of the intervention, by excluding its social effects. We will therefore undertake two kinds of analysis with regard to cluster effects: one where we consider the assumptions of independence to be upheld and one where we explore the effects of violation of these assumptions*.*

Despite these considerable challenges, Future Streets offers a unique opportunity to add new and important knowledge to the urgently needed international evidence base about how we reshape cities for health, equity and environmental sustainability.

## Abbreviations

CAS: Crash analysis system
CLD: Causal loop diagram
HbA1c: Glycated haemoglobin
IPAQ-LF: International physical activity questionnaire-long form
NO_2_: Nitrogen dioxide
NZ: New Zealand
NZTA: New Zealand Transport Agency
RTI: Road traffic injury
SER: Self-explaining roads
WHO: World Health Organisation
